# Supplementary material for: Evolution, structure and function of L-cysteine desulfidase, an enzyme involved in sulfur metabolism in the methanogenic archeon Methanococcus maripaludis
Source: Commun Biol. 2025 Nov 25;8:1667. doi: 10.1038/s42003-025-09053-0 (PMC12647591; doi:10.1038/s42003-025-09053-0)
Supplement: Supplementary file 2 — Supplementary Information [file 42003_2025_9053_MOESM2_ESM.pdf]

Supplementary Material

Evolution, structure and function of L-cysteine desulfidase, an enzyme involved in sulfur metabolism in the methanogenic archeon *Methanococcus maripaludis*

Sylvain Gervason, Paolo Zecchin, Elliot B. Shelton, Nisha He, Ludovic Pecqueur, Pierre Simon Garcia, Taiwo Akinyemi, Nadia Touati, Ornella Bimai, Christophe Velours, Jean-Luc Ravanat, Bruno Faivre, William B. Whitman, Marc Fontecave, Béatrice Golinelli-Pimpaneau

Supplementary data online:

Supplementary Data 1: CyuA.fasta (full dataset of CyuA sequences)
Supplementary Data 2: CyuA_SdaAB.fasta (Alignment of CyuA and SdaA/B sequences)
Supplementary Data 3: CyuA_SdaAB_Methyl-accepting-chemotaxis-proteins.fasta (Alignment of CyuA, SdaA/B and methyl-accepting chemotaxis proteins sequences)
Supplementary Data 4: CyuA_SdaAB_trimmed.phy (Trimmed alignment of sequences of CyuA and SdaA/B, to open with *aliview* https://ormbunkar.se/aliview/)
Supplementary Data 5: CyuA_SdaAB_Methyl-accepting-chemotaxis-proteins_trimmed.phy (Trimmed alignment of sequences of CyuA, SdaA/B and methyl-accepting chemotaxis proteins, to open with *aliview* https://ormbunkar.se/aliview/)
Supplementary Data 6: CyuA_SdaAB.tree (rooted phylogenetic tree of CyuA and SdaA/B to open with *Figtree* http://tree.bio.ed.ac.uk/software/figtree/)
Supplementary Data 7: CyuA_SdaAB_Methyl-accepting-chemotaxis-proteins_trimmed.tree (Phylogenetic tree of CyuA, SdaA/B and methyl-accepting chemotaxis proteins to open with *Figtree* http://tree.bio.ed.ac.uk/software/figtree/)
Supplementary Data 8: CSD.fasta (full dataset of CSD sequences)
Supplementary Data 9: Raw-data for Figure 2A and Figure 2B
Supplementary Data 10: Raw-data for Figure 2C
Supplementary Data 11: Raw data for Figure 3 and Supplementary Figure 8.
Supplementary Data 12: Raw data for Supplementary Figure 9.

Supplementary Tables 1 to 4
Supplementary Figures 1 to 15

Supplementary Table 1. Abundance of representative proteins and transcripts in *M. maripaludis*

proteins	Genetic loci	Proteome spectral counts [a]	Transcript abundance [b]	n[c]
Methyl-coenzyme M methylreductase	MMP1555, 1558, 1559	46.0 ± 26.9	16300 ± 900	3
heterodisulfide reductase	MMP1053, 1054	12.1 ± 0.6	1530 ± 420	2
CO dehydrogenase	MMP0980-0985	2.6 ± 1.0	535 ± 183	6
tryptophan operon	MMP1002-1008	1.2 ± 0.6	91 ± 31	7
cysteine desulfidase	MMP1468	0.3	30	1

[a] From (21). Proteome of strain S2 grown on H_2 + CO_2 in the presence of 2.8 mM cysteine and 3.2 mM sodium sulfide. Protein levels were estimated by the ratio of the number of peptides detected/ORF molecular weight.

[b] From (22). Transcripts abundance of strain S0001 grown on formate in the presence of 3.2 mM cysteine and 2.1 mM sodium sulfide are indicated as Fragments Per Kilobase of transcript per Million fragments mapped.

[c] The number of proteins or ORFs used to calculate the standard deviation

Supplementary Table 2: The effect of sulfide and L-cysteine concentrations on growth rates, final cell yields, and lag times of *M. maripaludis* S2 and Δ*mmp1468* strains.

Low inoculum: 10^5 cells per 5 mL culture

Strain	Na_2S (mM)	L-cysteine (mM)	Growth Rate (h^{-1})[a] (doubling time in h)	Final OD_{600}	Lag Time (h)	*p*-value[b]: Growth Rate / Final OD_{600} / Lag Time
**S2**	2	0	0.32 ± 0.004 (2.2)	1.04	19	< 0.0001 / < 0.0001 / < 0.0001
Δmmp1468	2	0	0.23 ± 0.01 (3)	0.84	26	
**S2**	2	1	0.33 ± 0.02 (2.1)	1.05	18	0.002 / < 0.0001 / 0.0002
Δmmp1468	2	1	0.21 ± 0.01 (3.3)	0.77	33	
**S2**	2	5	0.16 ± 0.09 (2.2)	1.08	28	N.S. / < 0.0001 / 0.0008
Δmmp1468	2	5	0.13 ± 0.09 (5.3)	0.76	73	
**S2**	2	10	0.16 ± 0.01 (4.3)	0.98	42	0.003 / 0.004 / 0.002
Δmmp1468	2	10	0.066 ± 0.009 (11)	0.52	130	
**S2**	0.4	0	0.19 ± 0.002 (3.6)	0.73	25	< 0.0001 / < 0.0001 / 0.04
Δmmp1468	0.4	0	0.13 ± 0.009 (5.3)	0.58	32	
**S2**	0.4	1	0.22 ± 0.01 (3.2)	0.77	25	0.0004 / < 0.0001 / 0.002
Δmmp1468	0.4	1	0.14 ± 0.004 (5.0)	0.56	35	
**S2**	0.4	5	0.16 ± 0.03 (4.3)	0.77	47	0.004 / < 0.0001 / 0.0001
Δmmp1468	0.4	5	0.04 ± 0.009 (17)	0.17	130	
**S2**	0.4	10	0.07 ± 0.03 (9.9)	0.29	140	N.D. / N.D. / N.D.
Δmmp1468	0.4	10	N.D.	N.D.	N.D.	

High inoculum: 10^7 cells per 5 mL culture

Strain	Na_2S (mM)	L-cysteine (mM)	Growth Rate (h^{-1})[a] (doubling time in h)	Final OD_{600}	Lag Time (h)	*p*-value[b]: Growth Rate / Final OD_{600} / Lag Time
**S2**	0	0	N.D.	0.018	N.D.	N.D. / N.S. / N.D.
Δmmp1468	0	0	N.D.	0.027	N.D.	
**S2**	0.1	0	N.D.	0.31	< 16	N.D. / N.S. / N.D.
Δmmp1468	0.1	0	0.057 ± 0.005 (12)	0.32	16	
**S2**	0	1	N.D.	0.027	N.D.	N.D. / 0.02 / N.D.
Δmmp1468	0	1	N.D.	0.043	N.D.	
**S2**	0	2	N.D.	0.059	N.D.	N.D. / 0.002 / N.D.
Δmmp1468	0	2	N.D.	0.032	N.D.	
**S2**	0	5	0.036 ± 0.01 (19)	0.30	440	N.D. / < 0.0001 / N.D.
Δmmp1468	0	5	N.D.	N.D.	N.D.	
**S2**	0	10	0.027 ± 0.002 (26)	0.38	230	N.D. / < 0.0001 / N.D.
Δmmp1468	0	10	N.D.	N.D.	N.D.	

N.D. = Not determined.
N.S. = Not significant
[a]The mean and standard error of the mean (SEM) were calculated from the highest growth rate for each tube within a growth condition.
[b]If *p*-value < 0.0001, entered as < 0.0001. If other value, then rounded to nearest single numeral greater than 0.

Supplementary Table 3: Fe content after cluster reconstitution of wild-type MmCyuA and variants.

Enzyme	number of iron atoms per monomer*
holo-CyuA	3.3 ± 0.2
apo-CyuA	0 ± 0.2
holo-C289A-CyuA	3.6 ± 0.1
holo-C23A-CyuA	3.6 ± 0.2
holo-E25A-CyuA	3.8 ± 0.2
holo-E25D-CyuA	3.2 ± 0.3
holo-E25Q-CyuA	3.4 ± 0.2

*The data shown are mean values based on 3 different experiments.

Supplementary Table 4. Primers used for deletion of the *mmp1468* gene.

A. Primers for UP PCR.

Primers	TM (°C)[§]	Sequences (5'-3')	Product(bp)
Up_FP	52	TGCACCAGCACCAAGAG	735
Up_RP	55	GGGAAAGGCCTGACTGGCCATCCATAATATCAACTAAATCTTTAG	

B. Primers for DOWN PCR.

Primers	TM (°C)[§]	Sequences (5'-3')	Product(bp)
Down_FP	53	GGGAAAGGCCAAGGTGGCCAATTTAATATTTTTTCTATTTTATTCTTCG	594
Down_RP	53	ATTACCGGTGAAGAATACG	

C. Standardized primers for p5L vectors.

Primers	TM (°C)[§]	Sequences (5'-3')[¶]	Product(kb)
RE-F(SfiI-a)	65	GGGAAA**GGCCAGTCAGGCC**GAAAGGGGGATGTGCTGC	1.67
RE-R(SfiI-b)	66	GGGAAA**GGCCACCTTGGCC**TGGAACTTGATTTAAATTTAGTGGAATTC	

[§]TM values are calculated by the NEB Tm Calculator https://tmcalculator.neb.com/ with Q5 High-Fidelity DNA Polymerase as the polymerase kit.
[¶]The SfiI sites are indicated in bold.

Supplementary Figures

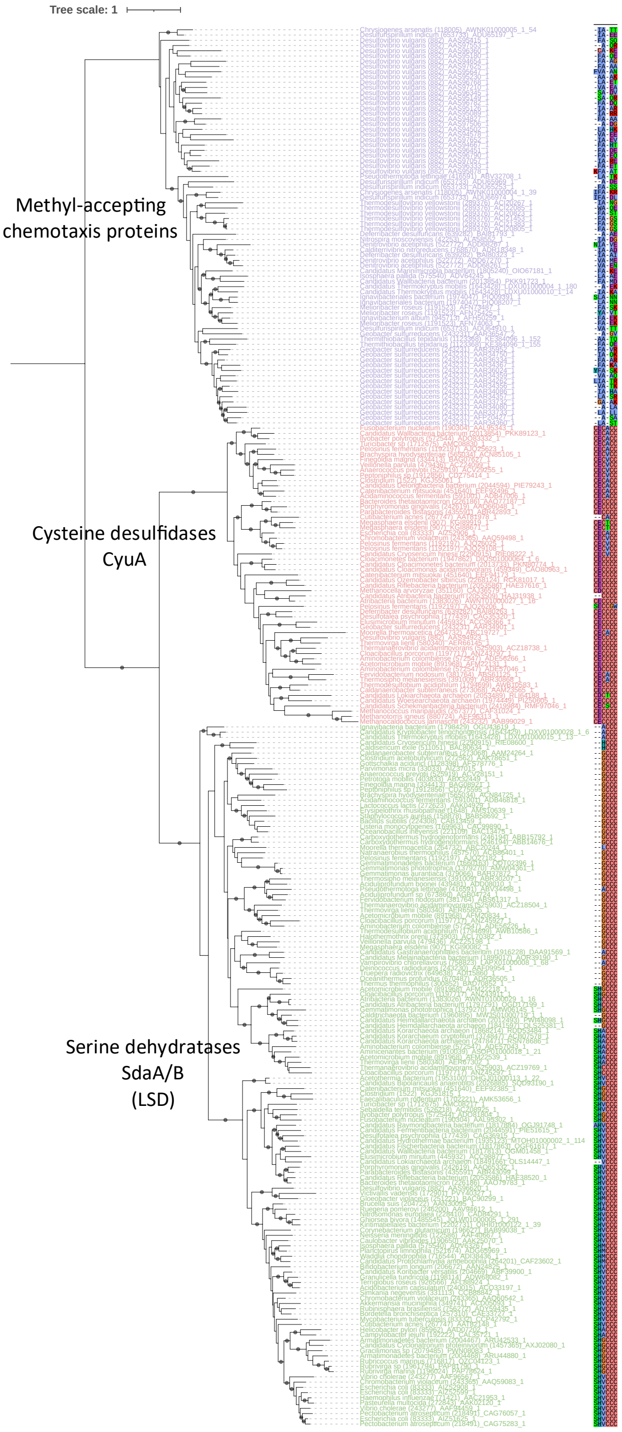

Supplementary Figure 1. Phylogeny of CyuA homologues (267 sequences, 230 amino-acid positions, IQ-TREE, LG+R8). The dots at branches correspond to ultrafast bootstrap values ≥ 95%. The scale bar represents the average number of substitutions per site. The leaves are colored according to the delineation of the 3 subfamilies, methyl-accepting chemotaxis protein, CyuA and SdaAB. The name of the organism is followed by the taxonomic ID (in parenthesis) and the protein accession number. The conservation of the six residues mentioned in the text is shown, with the numbers corresponding to the positions in the sequence of MmCyuA (23, 25, 287, 289, 329, 336). The sequence alignment files are available as Supplementary Data 1 to 5 and the tree file is available as Supplementary Data 7.

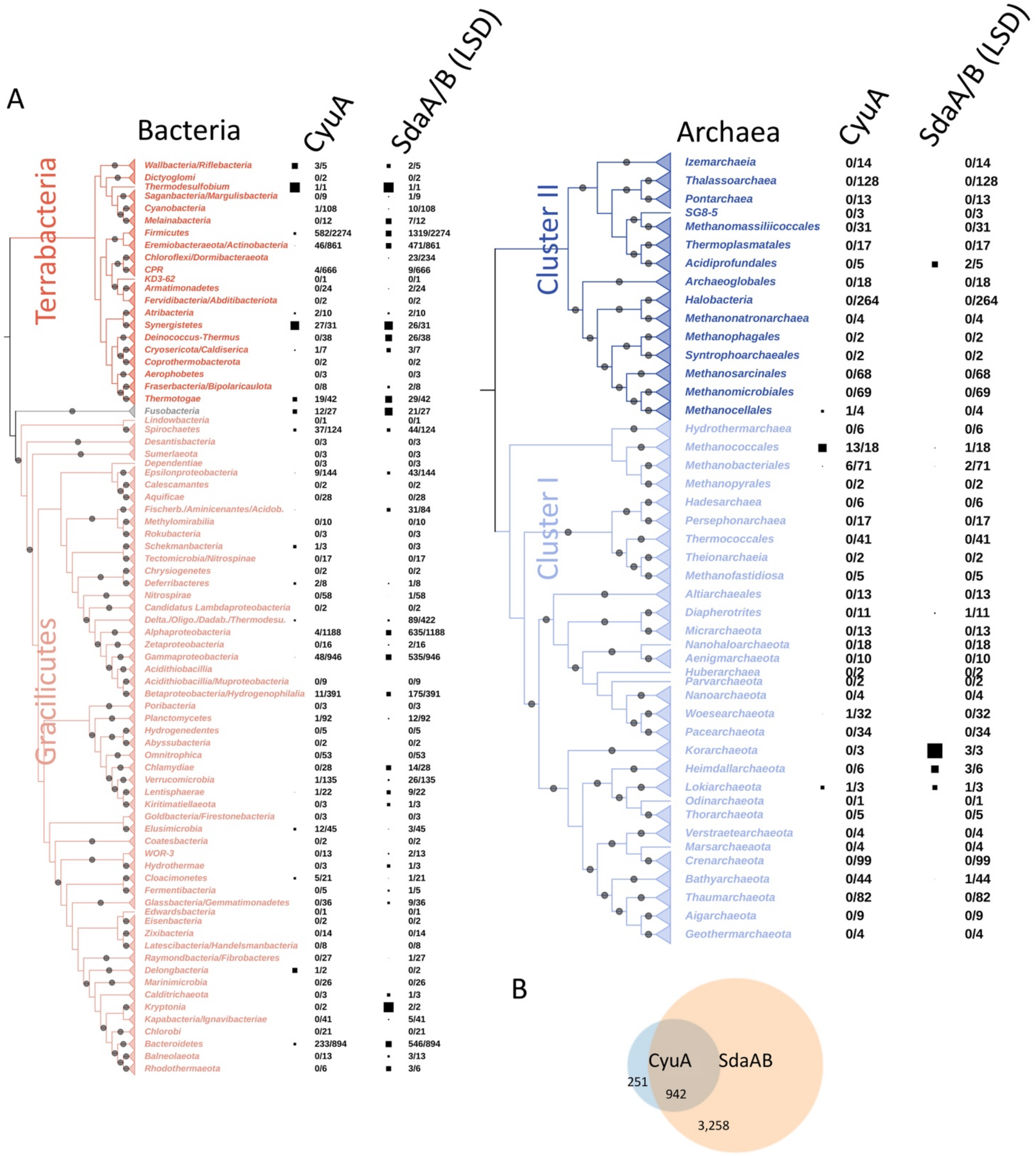

Supplementary Figure 2: **CyuA is unevenly distributed among Prokaryotes**. **A** Taxonomic distribution of CyuA and SdaAB in Bacteria and Archaea. The phylogenetic trees of Archaea and Bacteria were adapted from Garcia PS*, et al. Nat Ecol Evol* 6, 1564-1572 (2022) see Material & Methods. The dots at the branches correspond to ultrafast bootstrap values greater than 95%. For each phylum/clade, the proportion of genomes possessing at least one copy of CyuA/SdaAB is indicated, with the size of the black squares proportional to this ratio. The colors of clades correspond to major groups of Bacteria (Light red: Gracilicutes, Dark red: Terrabacteria, Grey: Fusobacteria, which does not belong to either group) and Archaea (Light blue: Cluster I, Dark blue: Cluster II). **B** Venn diagram of genomes possessing CyuA, SdaA/B or both. The number of genomes is indicated.

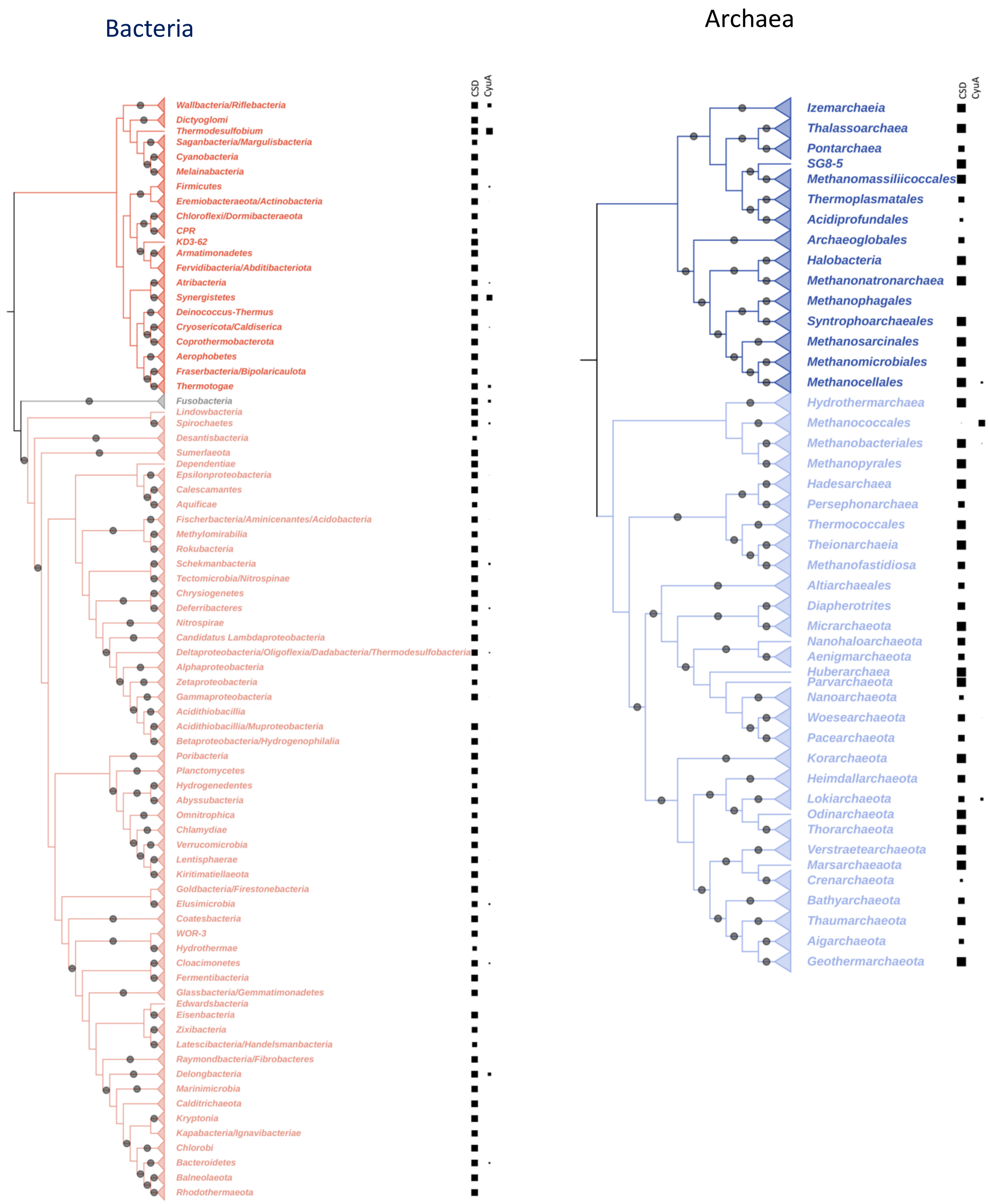

Supplementary Figure 3. Taxonomic distribution of cysteine desulfurase (CSD) and CyuA in Bacteria and Archaea. The phylogenetic trees of Archaea and Bacteria and taxonomic distribution of CSDs have been adapted from Garcia PS, et al. Nat Ecol Evol 6, 1564-1572 (2022) (see Material & Methods). The dots at the branches correspond to ultrafast bootstrap values greater than 95%. For each phylum/clade, the proportion of genomes possessing at least one copy of CSD/CyuA is indicated by black squares proportional to this ratio. The colors of clades correspond to major groups of Bacteria (Light red: Gracilicutes, Dark red: Terrabacteria, Grey: Fusobacteria, which does not belong to either group) and Archaea (Light blue: Cluster I, Dark blue: Cluster II). The sequence alignment file of CSD is available as Supplementary Data 8.

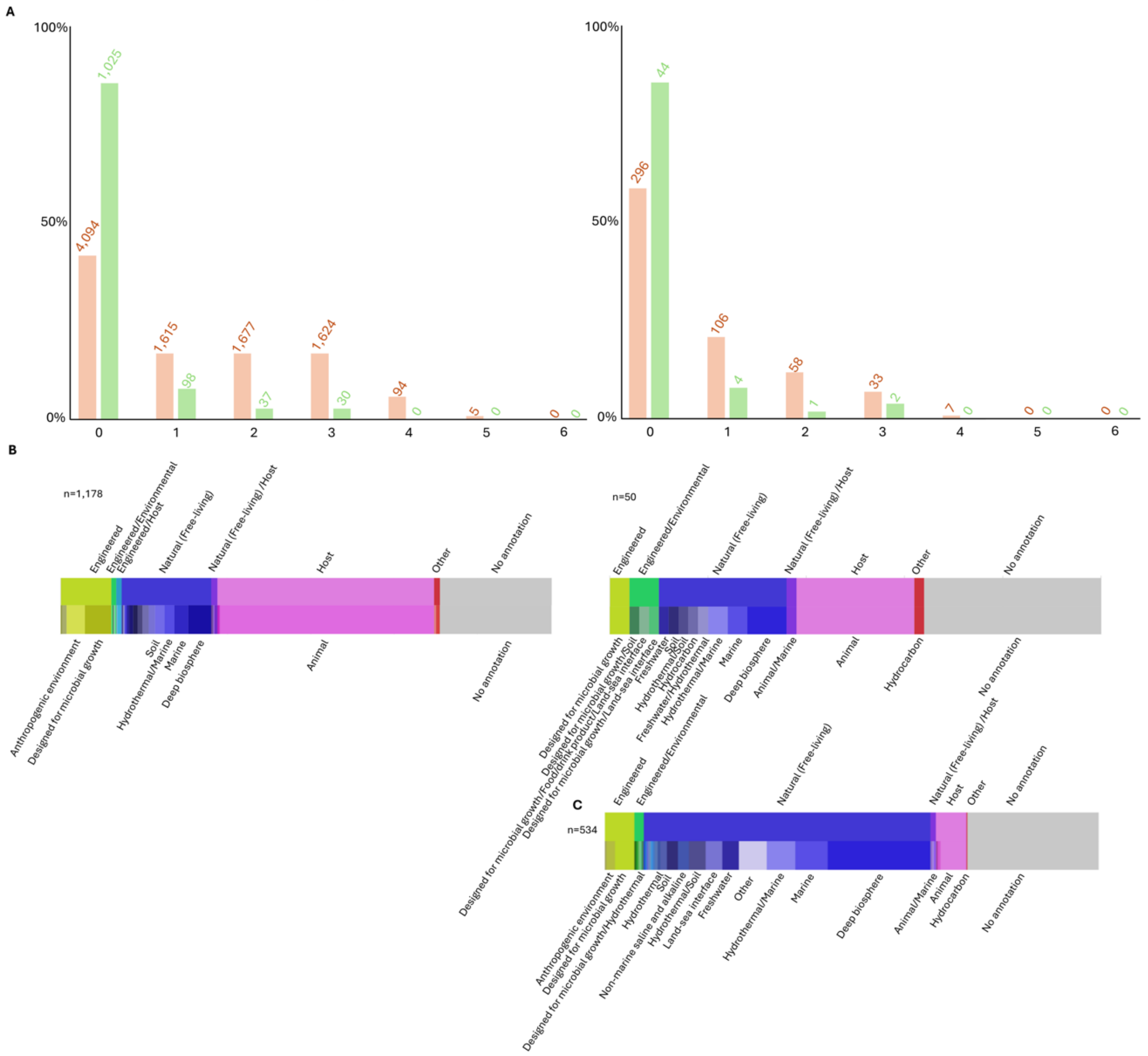

Supplementary Figure 4. A. Distribution of the number of oxygen exposure markers present in genomes. Salmon: genomes without CyuA, green: genomes possessing CyuA. Left: analysis of the entire database of 10,864 proteomes of prokaryotes. Right: analysis of the subsampled database of 551 representative proteomes of prokaryotes. **B**. Distribution of the different ecological niches of organisms possessing CyuA. Left: analysis of the entire database of 10,864 proteomes of prokaryotes (1,178 genomes with CyuA). Right: analysis of the subsampled database of 551 representative proteomes of prokaryotes (50 genomes with CyuA). The number of proteomes possessing CyuA that have been analyzed is indicated. The environments are categorized by two layers, a global category (up) and sub-categories with specific niches (bottom). Only the major sub-categories are indicated for the entire database. **C** Distribution of the different ecological niches of all organisms from the subsampled database (17 genomes were not found in the METACAT database).

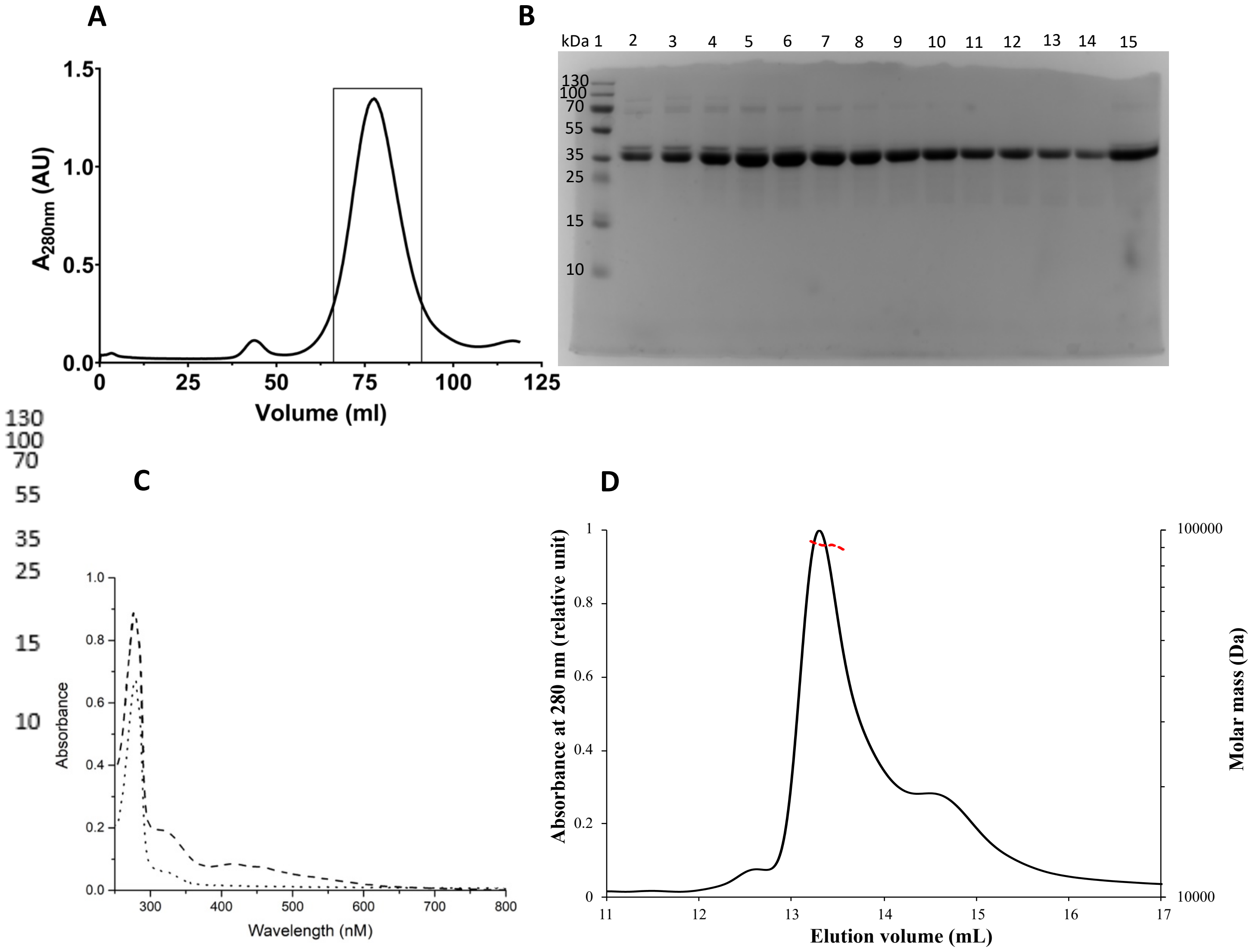

Supplementary Figure 5. Purification and characterization of as-purified MmCyuA. **A** Purification of recombinant MmCyuA under aerobic conditions on a Sephacryl S200 gel filtration column in 25 mM HEPES pH 7.5, 200 mM NaCl, 5 mM DTT. The framed fractions were pooled. **B** 14% SDS-PAGE gel of as-purified MmCyuA. Lane 1: standards; lanes 2 to 14: fractions along the column; lane 15: pooled fractions. **C** UV-visible spectra of as-purified MmCyuA (dashed line) and apo-MmCyuA (dotted line). The spectra were recorded with 40 µM protein in 25 mM HEPES pH 7.5, 200 mM NaCl. **D** SEC-MALS analysis of apo-MmCyuA. Apo-MmCyuA (2mg/ml) was loaded onto a Superdex 200 10/300 GL increase column equilibrated with 25 mM HEPES pH 7.5, 150 mM NaCl. The absorbance at 280 nm (black line) and weighted-average molar mass (dotted red line) are plotted as a function of the elution volume. The first main peak corresponds to a molar mass of 78.6 ± 2.1 kDa, indicating a dimer (theoretical mass of monomer: 43.7 kDa).

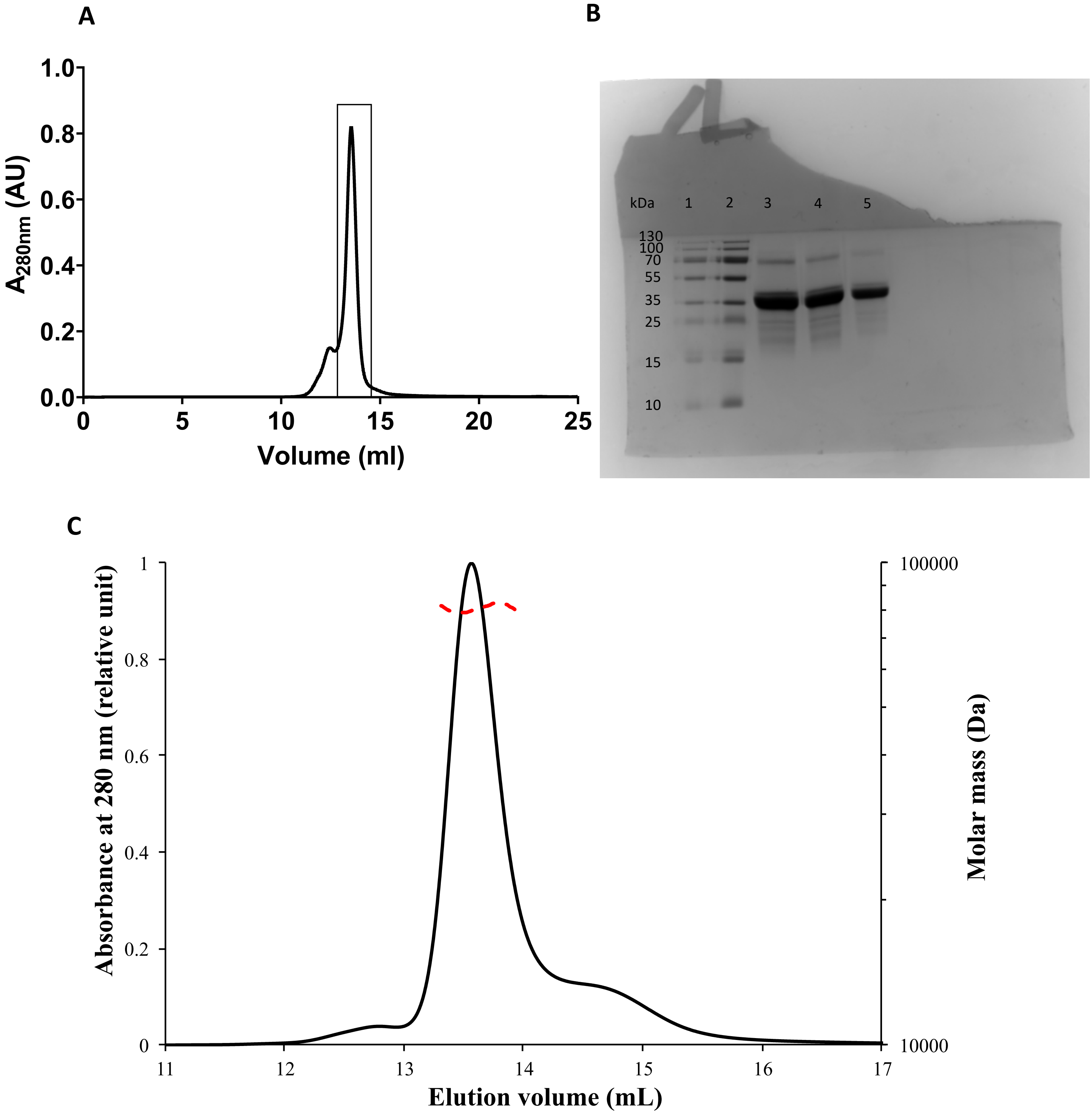

Supplementary Figure 6. Purification and characterization of holo-MmCyuA. **A** Chromatographic profile of holo-MmCyuA on a Sephacryl S200 gel filtration column. **B** 14% SDS-PAGE gel of holo-MmCyuA. Lanes 1 and 2: standards; lanes 3 to 5: fractions along the column. **C** SEC-MALS analysis of holo-MmCyuA. Holo-MmCyuA (2mg/ml) was loaded onto a Superdex 200 10/300 GL increase column equilibrated with 25 mM HEPES pH 7.5, 150 mM NaCl. The absorbance at 280 nm (black line) and weighted-average molar mass (dotted red line) were plotted as a function of the elution volume. The first main peak corresponded to a molar mass of 80.8 ± 2.1 kDa, indicating a dimer (theoretical mass of monomer: 43.7 kDa).

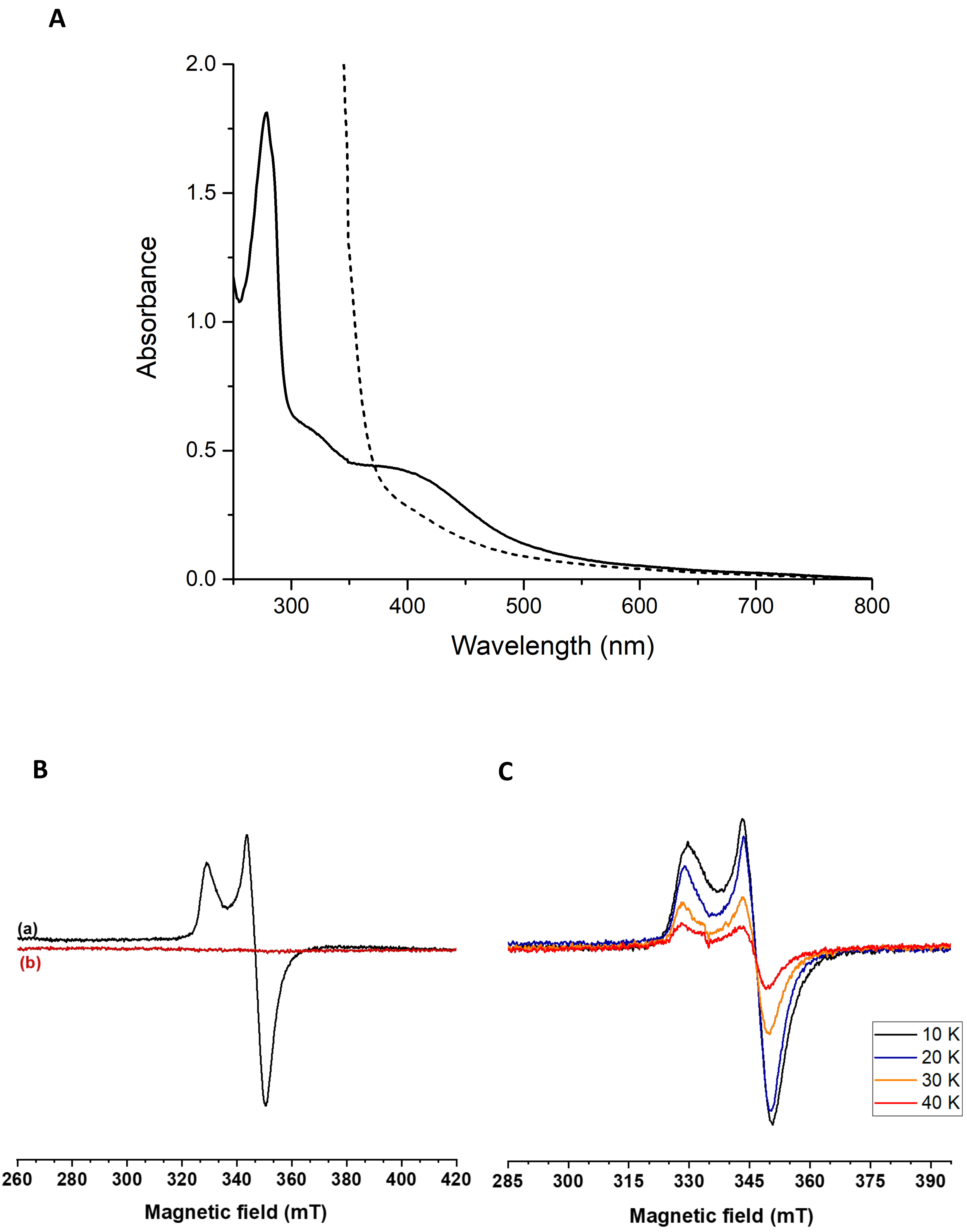

Supplementary Figure 7. Spectroscopic characterization of holo-MmCyuA. **A** UV-visible spectra of holo-MmCyuA (thick line) and reduced holo-MmCyuA (dashed line) after incubation with 1 mM dithionite for 20 min. The spectra were recorded with 40 µM protein in 25 mM HEPES pH 7.5, 200 mM NaCl. **B** X-band EPR spectrum of MmCyuA. CW-EPR spectrum of holo-MmCyuA (200 µM) in 25 mM HEPES pH 7.5, 200 mM NaCl, before (in red) and after (in black) reduction with 5 mM dithionite for 30 min at 20°C. **C** Variation with temperature of the X-band CW-EPR spectrum of reduced MmCyuA at 2 mW.

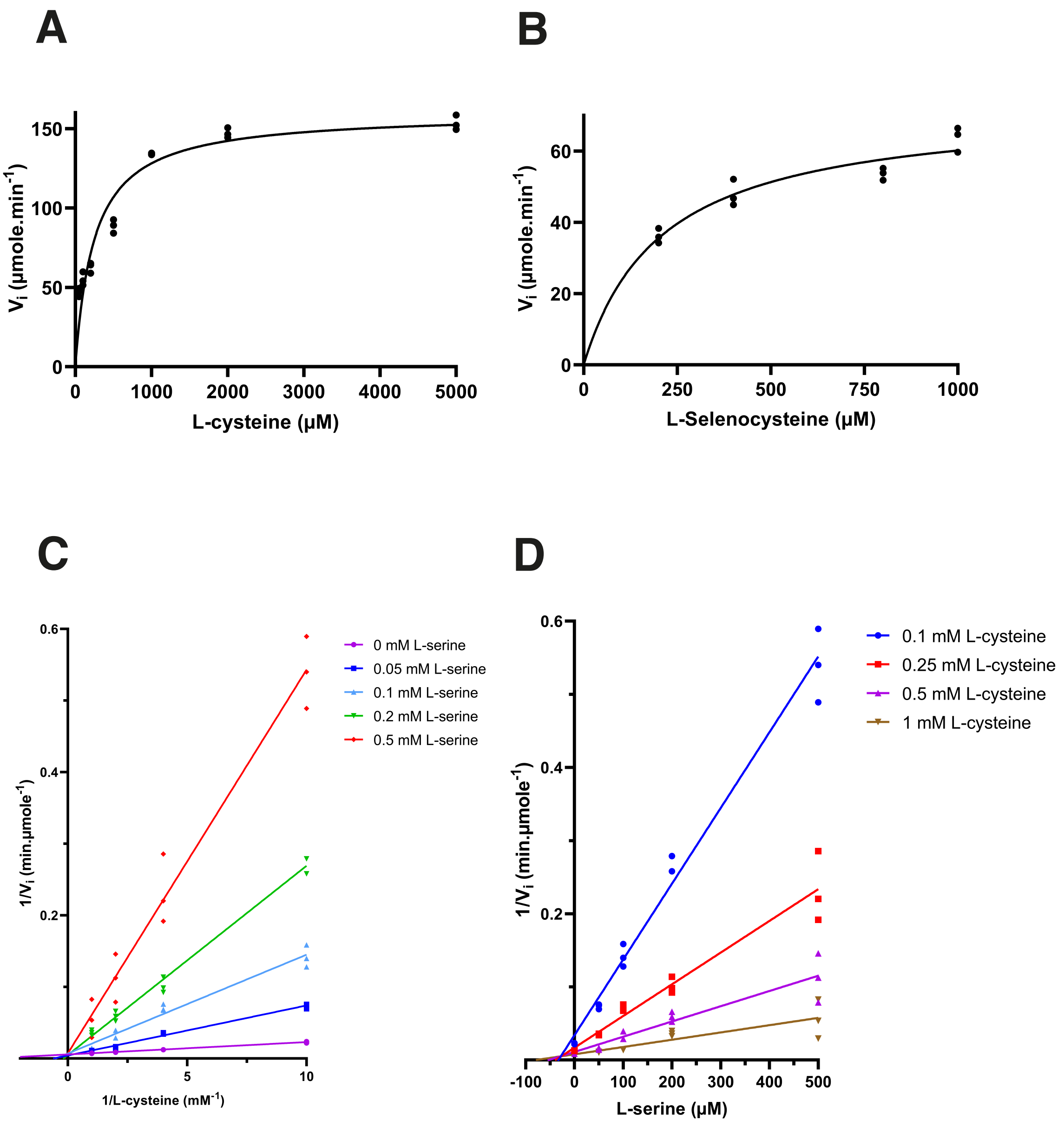

Supplementary Figure 8. Determination of the kinetic parameters for cysteine and selenocysteine as substrates, and serine as an inhibitor of holo-MmCyuA. Incubations were performed with 1 µM holo-MmCyuA in 25 mM HEPES pH 7.5, 0.15 M NaCl. The data shown are mean values based on 3 different experiments. **A and B** Determination of the Michaelis constants for L-cysteine (A) or L-selenocysteine (B) as substrates by fitting the data to the Michaelis-Menten equation by nonlinear regression using GraphPad. **C and D** Determination of the inhibition constant for L-serine. **C** The Lineweaver-Burk plots of the reciprocal of initial rate versus the reciprocal of cysteine concentration at different serine concentrations intercept on the y-axis, showing competitive inhibition. **D** Dixon plot showing the second plot of slopes as a function of serine concentration. The curves intercept at [I]= $-K_i$.

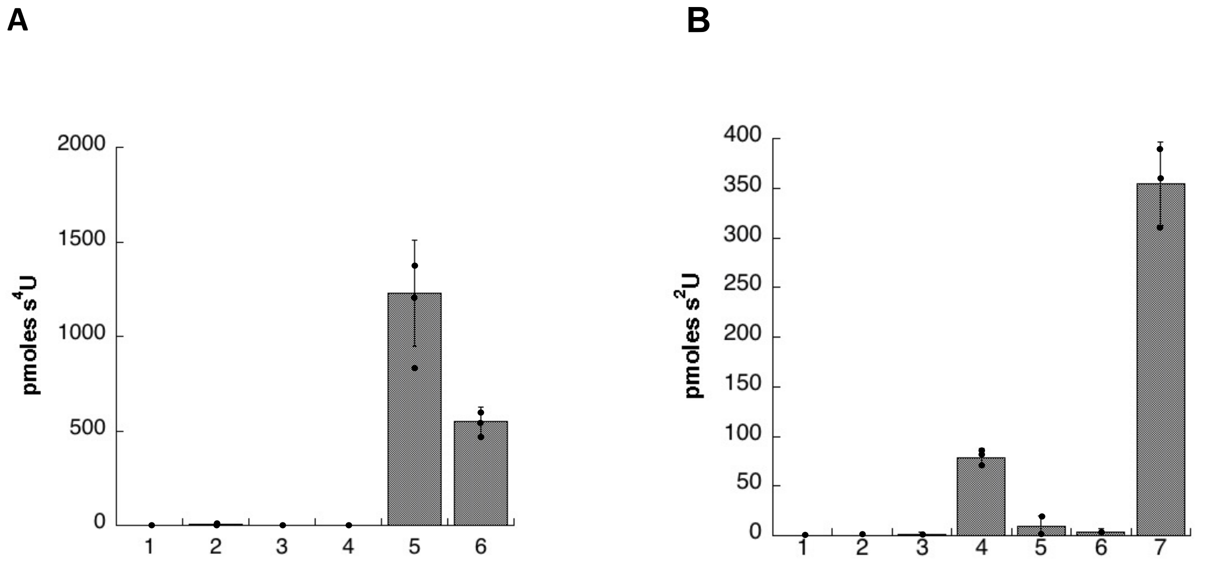

Supplementary Figure 9. holo-MmCyuA can provide sulfur from L-cysteine to tRNA thiolases *in vitro*. Conditions for s^{4}U- and s^{2}U-tRNA formation by MmTtuI and MmNcsA monitored by HPLC-coupled MS/MS after tRNA digestion. The data shown are mean values based on 3 different experiments. **A** apo- or holo-MmCyuA (10 µM) was incubated with apo- or holo-MmTtuI (1 µM) and tRNA$^{Lys}_{UUU}$ transcript (15 µM), in the absence or presence of cysteine (1 mM) or Na_2S (1 mM) as the sulfur source. (1) tRNA alone. (2) apo-MmTtuI, holo-MmCyuA and cysteine. (3) holo-MmTtuI, apo-MmCyuA and cysteine. (4) holo-MmTtuI, holo-MmCyuA in the absence of cysteine. (5) holo-MmTtuI, holo-MmCyuA and cysteine. (6) holo-MmTtuI and Na_2S. **B** apo- or holo-MmCyuA (2 µM) was incubated with apo or holo-MmTtuI (1 µM) and tRNA$^{Lys}_{UUU}$ transcript (20 µM) in the presence or absence of cysteine (0.25 mM) or Na_2S (0.25 mM) as the sulfur source. (1) tRNA alone. (2) apo-MmNcsA and Na_2S. (3) holo-MmNcsA and no sulfur source. (4) holo-MmNcsA and Na_2S. (5) holo-MmNcsA, apo-MmCyuA and cysteine. (6) apo-MmNcsA, holo-MmCyuA and cysteine. (7) holo-MmNcsA, holo-MmCyuA and cysteine.

A

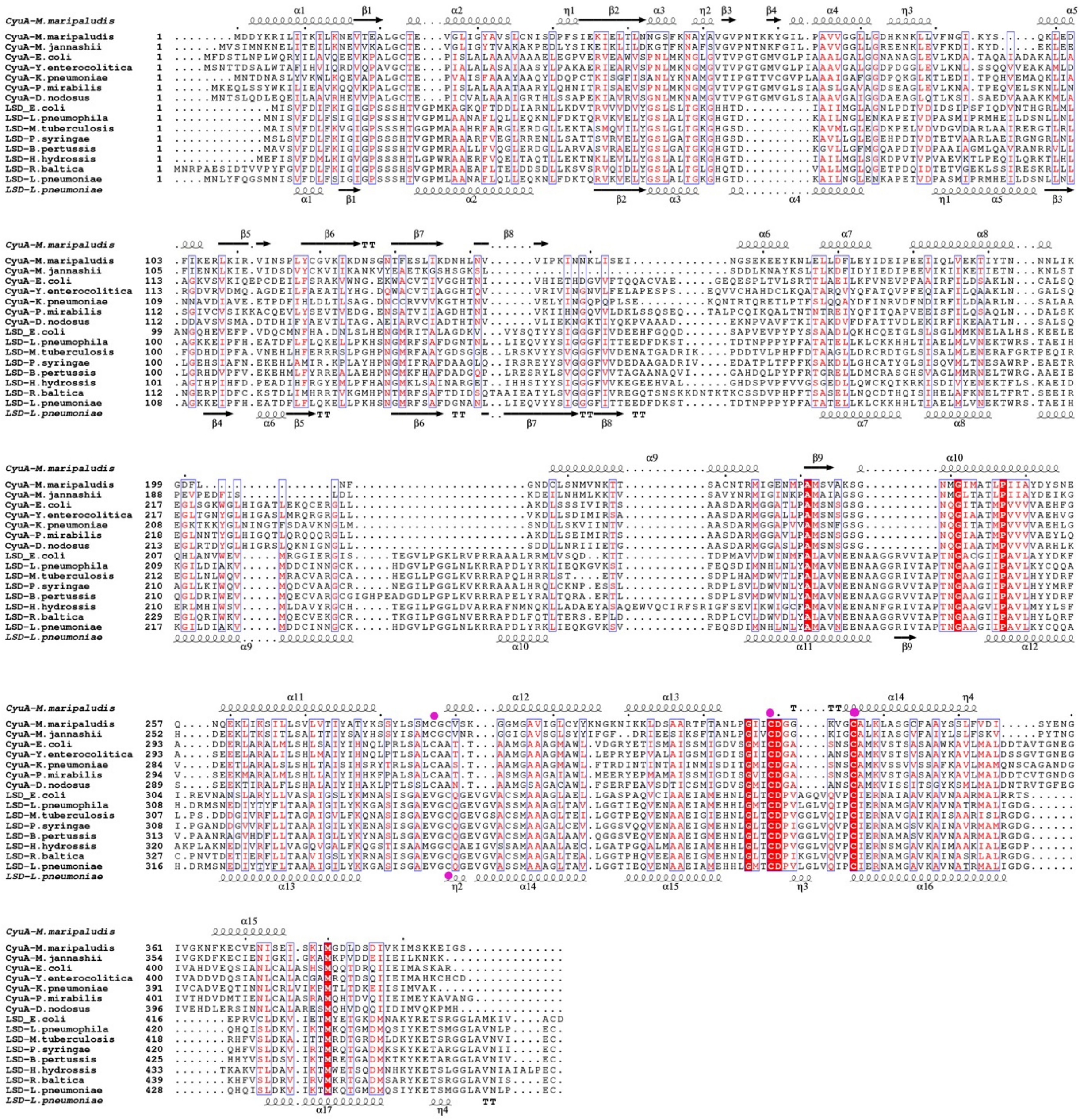
CyuA-M.maripaludis
CyuA-M.jannashii
CyuA-E.coli
CyuA-Y.enterocolitica
CyuA-K.pneumoniae
CyuA-P.mirabilis
CyuA-D.nodosus
LSD_E.coli
LSD-L.pneumophila
LSD-M.tuberculosis
LSD-P.syringae
LSD-B.pertussis
LSD-H.hydrossis
LSD-R.baltica
LSD-L.pneumoniae
LSD-L.pneumoniae

B

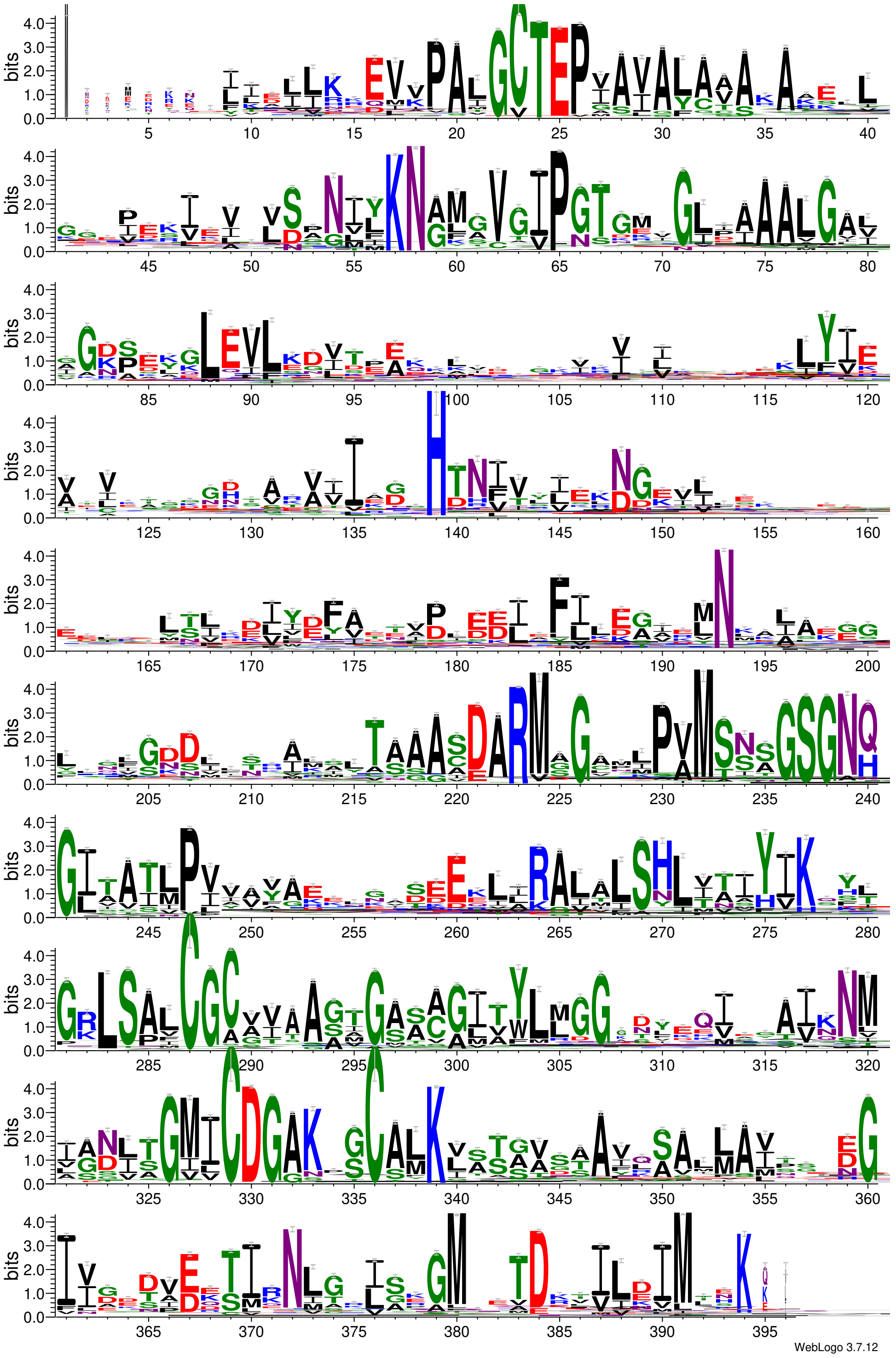

Supplementary Figure 10. Amino acid sequence alignment of several members of the CyuA and [FeS]-dependent LSD proteins. A Alignment of CyuA (28) and LSD sequences was performed with Clustal Omega (93) and rendered with *ESPript* (94). The secondary structure elements of MmCyuA and LpLSD are shown above and below the alignment, respectively. All enzymes contain three conserved cysteines (indicated as magenta spheres) that were shown to coordinate a [4Fe-4S] cluster in MmCyuA and LpLSD. **B** The mmseq2 alignment (1392 sequences) obtained with Colabfold (https://colab.research.google.com/github/sokrypton/ColabFold/blob/main/AlphaFold2) was filtered with *HHFilter* (95) using the following parameters: Min Cov 60% Max Id 90%. The resulting alignment was rendered with WebLogo (96).

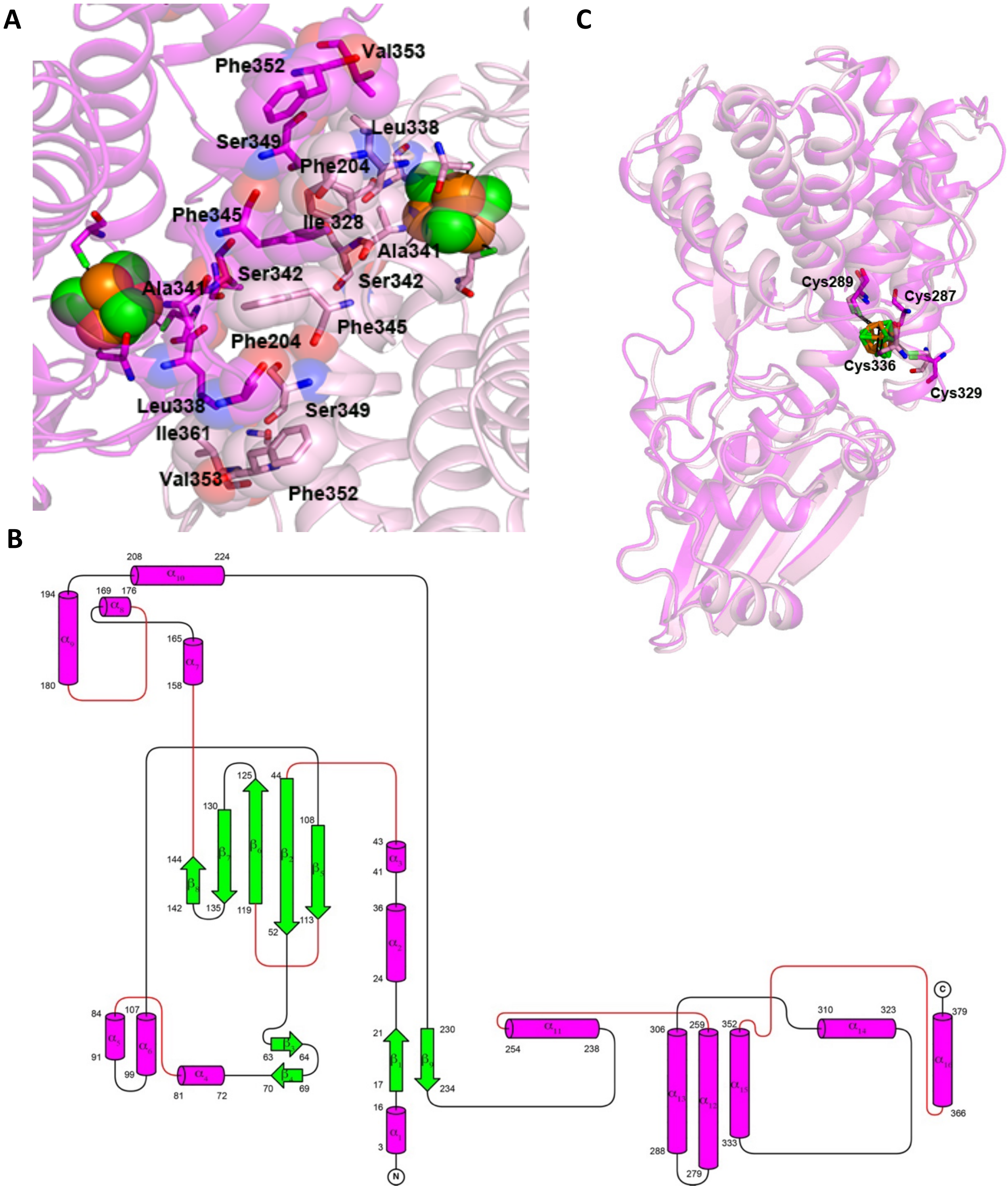

Supplementary Figure 11. Overall fold of MmCyuA and interface between two monomers in the holo-MmCyuA crystal structure. A Hydrophobic interactions at the dimer interface of the holo-MmCyuA crystal structure (same orientation as Figure 4A; monomer A in magenta, monomer B in pink). **B** Topology diagram calculated with TOPDRAW in CCP4 showing the arrangement of the α-helices and β-sheets in MmCyuA. **C** Superposition of the two molecules of the MmCyuA structure. Molecules A and B of the MmCyuA structure were superimposed in PYMOL with an rmsd of 0.74 Å for 301 aligned Cαs. The N-terminal domain (bottom) and the C-terminal catalytic domain (top) that binds the cluster (shown in stick representation) are linked by a flexible linker.

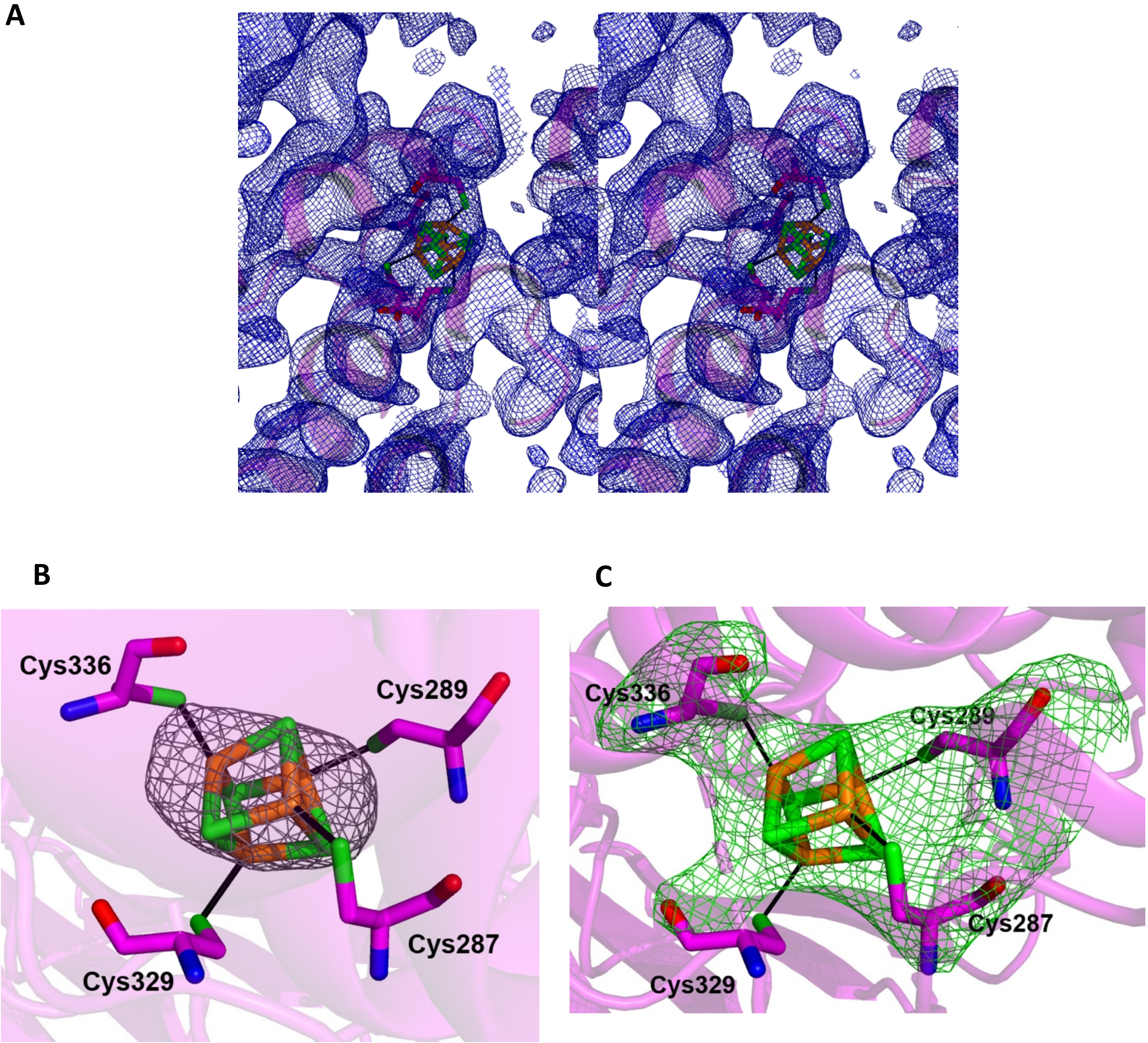

Supplementary Figure 12. Electron density maps of nonliganded wild-type holo-MmCyuA structure (molecule A). **A** Stereoview of the [4Fe-4S] binding site with a 2Fo-Fc map contoured at 1 σ (in blue). **B** Anomalous map contoured at 5 σ (in grey) for data collected near the K-edge of iron superimposed on the cluster site. **C** Fo-Fc map contoured at 3.5 σ (in green) omitting the cluster and the four chelating cysteines superimposed on the cluster site.

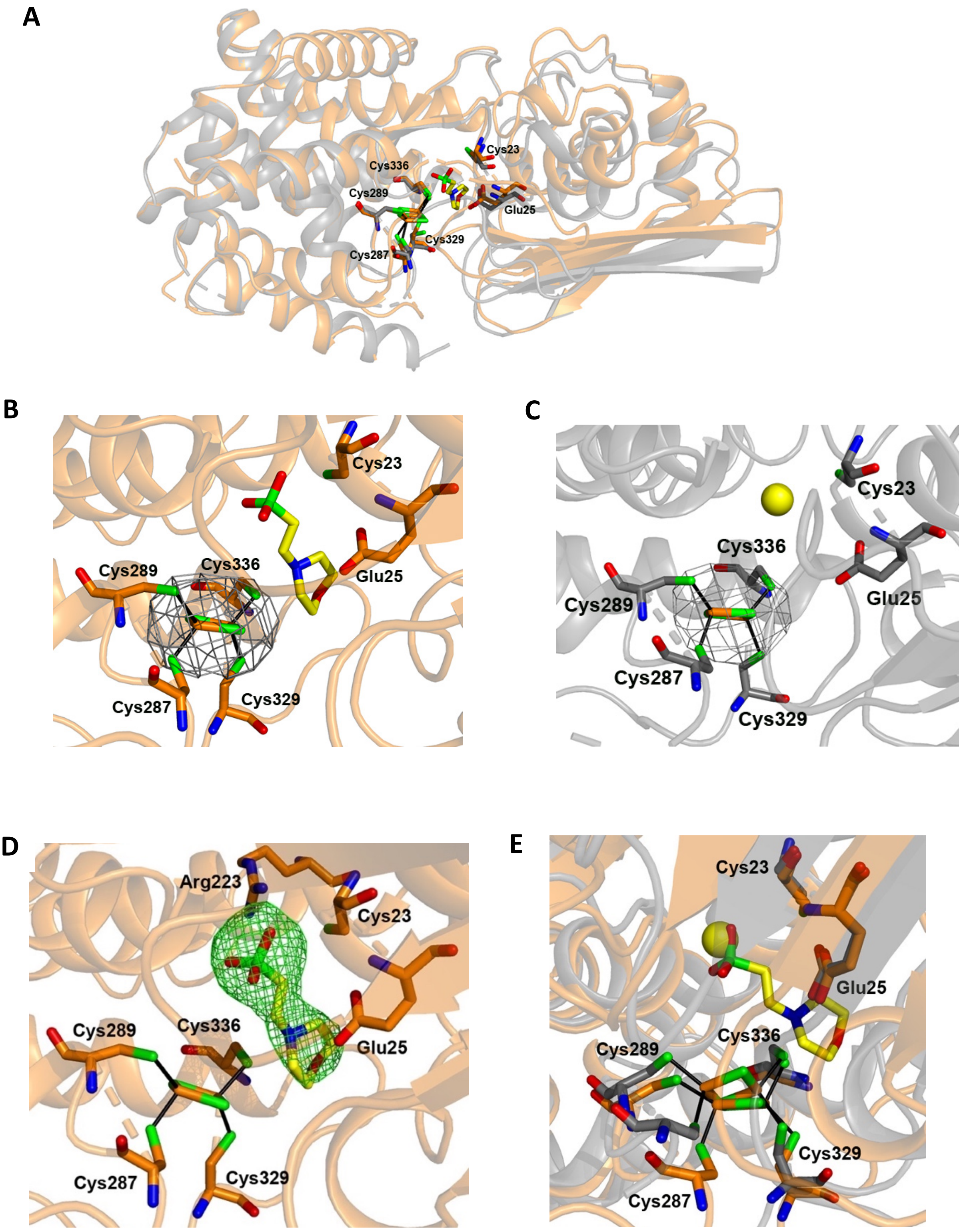

Supplementary Figure 13. Structure of [2Fe-2S]-MmCyuA in complex with MES. **A** Superposition of molecules A (in orange) and B (in grey) of [2Fe-2S]-MmCyuA in complex with MES. The C-terminal domains of molecules A and B (residues 157-378) were superimposed in PYMOL with an rmsd of 0.094 Å for 155 aligned Cαs. The MES ligand in molecule A is shown as yellow sticks. **B** Fo-Fc anomalous map (in grey) contoured at 5 σ centered on the active site of molecule A with MES in yellow sticks. **C** Fo-Fc anomalous map (in grey) contoured at 5 σ centered on the active site of molecule B, with chloride shown as a yellow sphere. **D** Fo-Fc map (in green) omitting the MES ligand contoured at 3 σ. **E** Superposition of the active sites of molecules A and B of the [2Fe-2S]-MmCyuA structure. The chloride ion in molecule B is represented as a yellow sphere.

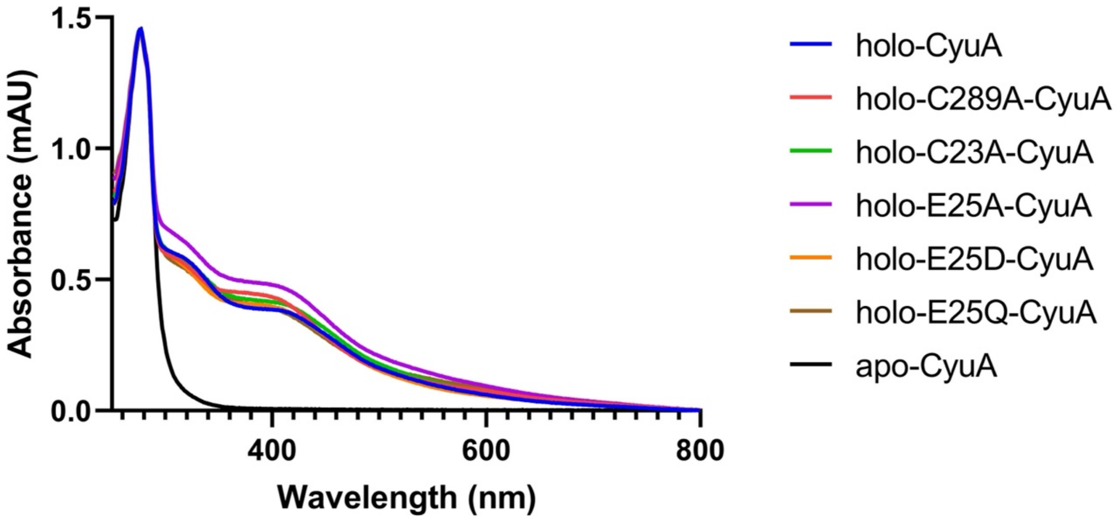

Supplementary Figure 14. Absorption spectra of MmCyuA mutants after cluster reconstitution.

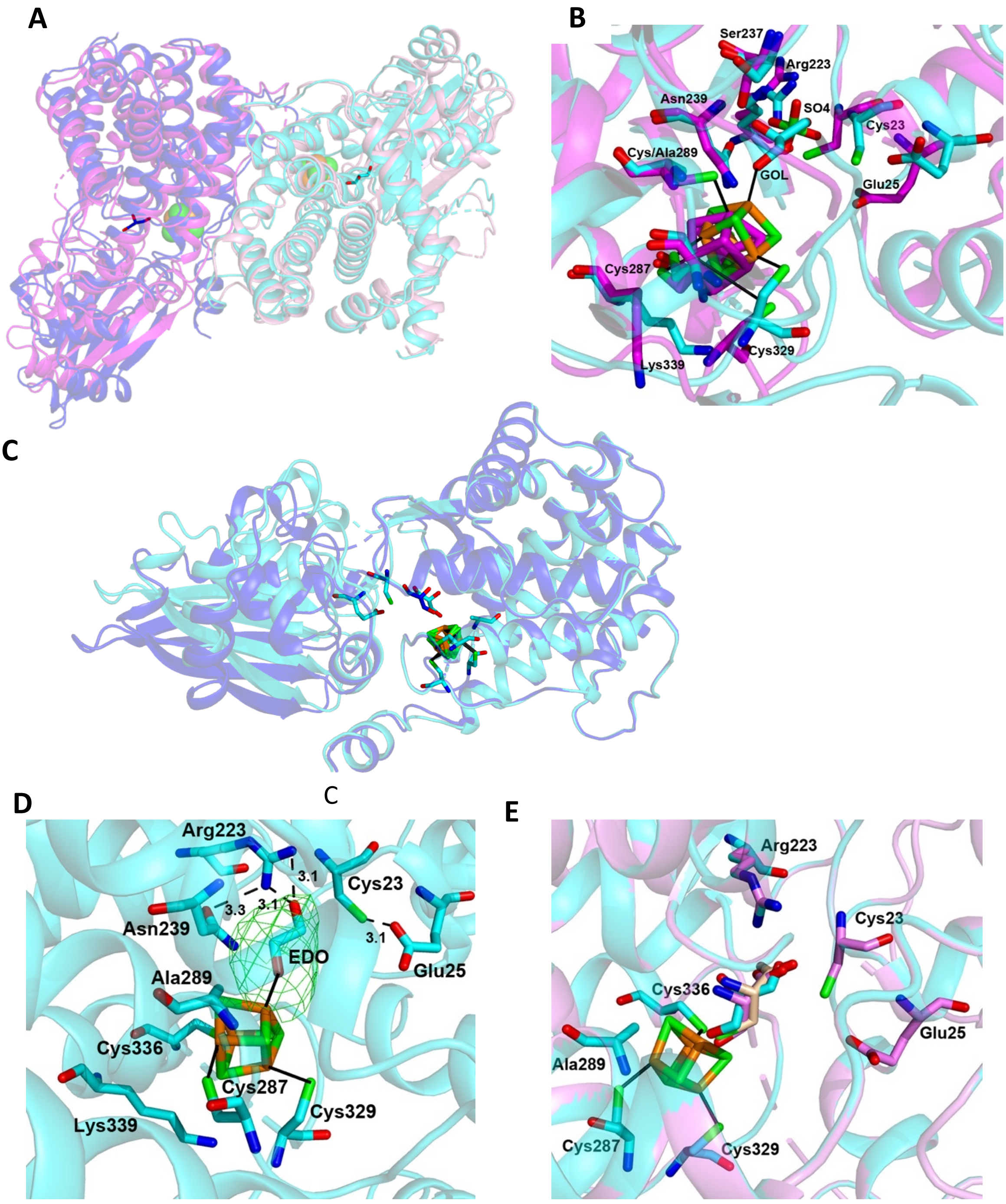

Supplementary Figure 15. Structure of holo-C289A-MmCyuA. A Superposition of the dimers of holo-MmCyuA alone (monomers in pink and magenta, clusters as spheres) and holo-C289A-MmCyuA + glycerol (monomers in cyan and blue green, glycerol as sticks). The C-terminal catalytic domains of molecules A of both crystal structures were superposed in PYMOL with a rmsd of 0.325 Å for 108 aligned Cαs. **B** Superposition of the active sites of holo-MmCyuA and holo-C289A-MmCyuA with bound glycerol. **C** The C-terminal catalytic domains of molecules A (in cyan) and B (in blue) of C289A-holo-MmCyuA with bound glycerol were superimposed in PYMOL with an rmsd of 0.165 Å for 130 aligned Cαs. The C-terminus is indicated by 'C'. **D** Active site structure of holo-C289A-MmCyuA with bound ethylene glycol (EDO). A Fobs-Fcal map omitting the ethylene glycol ligand (in green) is contoured at 3.5 σ. **E** Superposition of the models of the cysteine substrate and serine inhibitor bound to holo-C289A-MmCyuA with the crystal structure of glycerol-bound holo-C289A-MmCyuA.
