# Supplementary material for: Evolution, structure and function of L-cysteine desulfidase, an enzyme involved in sulfur metabolism in the methanogenic archeon Methanococcus maripaludis
Source: Commun Biol. 2025 Nov 25;8:1667. doi: 10.1038/s42003-025-09053-0 (PMC12647591; doi:10.1038/s42003-025-09053-0)
Supplement: Supplementary file 3 — Description of Additional Supplementary Files [file 42003_2025_9053_MOESM3_ESM.pdf]

Description of Additional Supplementary Files

File name- Supplementary Data 1
File description – CyuA.fasta (Full dataset of CyuA sequences)

File name- Supplementary Data 2
File description – CyuA_SdaAB.fasta (Alignment of CyuA and SdaA/B sequences)

File name- Supplementary Data 3
File description – CyuA_SdaAB_Methyl-accepting-chemotaxis-proteins.fasta (Alignment of CyuA, SdaA/B and methyl-accepting chemotaxis proteins sequences)

File name- Supplementary Data 4
File description – CyuA_SdaAB_trimmed.phy (Trimmed alignment of sequences of CyuA and SdaA/B, to open with aliview https://ormbunkar.se/aliview/)

File name- Supplementary Data 5
File description – CyuA_SdaAB_Methyl-accepting-chemotaxis-proteins_trimmed.phy (Trimmed alignment of sequences of CyuA, SdaA/B and methyl-accepting chemotaxis proteins, to open with aliview https://ormbunkar.se/aliview/)

File name- Supplementary Data 6
Filedescription– CyuA_SdaAB.tree (Rooted phylogenetic tree of CyuA and SdaA/B to open with Figtree http://tree.bio.ed.ac.uk/software/figtree/)

File name- Supplementary Data 7
File description – CyuA_SdaAB_Methyl-accepting-chemotaxis-proteins_trimmed.tree (Phylogenetic tree of CyuA, SdaA/B and methyl-accepting chemotaxis proteins to open with Figtree http://tree.bio.ed.ac.uk/software/figtree/)

File name- Supplementary Data 8
File description – CSD.fasta (Full dataset of CSD sequences)

File name- Supplementary Data 9
File description – Raw-data for Figure 2A and Figure 2B

File name- Supplementary Data 10
File description – Raw-data for Figure 2C

File name- Supplementary Data 11
File description – Raw data for Figure 3 and Supplementary Figure 8

File name- Supplementary Data 12
File description – Raw data for Supplementary Figure 9
